# Recognition of essential purines by the U1A protein

**DOI:** 10.1186/1471-2091-8-22

**Published:** 2007-11-02

**Authors:** Yulia Benitex, Anne M Baranger

**Affiliations:** 1 Department of Chemistry, Wesleyan University, Middletown, CT 06459, USA; 2 University of Illinois, Department of Chemistry, 600 South Mathews Avenue, Urbana, IL 61801, USA

## Abstract

**Background:**

The RNA recognition motif (RRM) is one of the largest families of RNA binding domains. The RRM is modulated so that individual proteins containing RRMs can specifically recognize RNA targets with diverse sequences and structures. Understanding the principles governing this specificity will be important for the rational modification and design of RRM-RNA complexes.

**Results:**

In this paper we have investigated the origins of specificity of the N terminal RRM of the U1A protein for stem loop 2 (SL2) of U1 snRNA by substituting modified bases for essential purines in SL2 RNA. In one series of modified bases, hydrogen bond donors and acceptors were replaced by aliphatic groups to probe the importance of these functional groups to binding. In a second series of modified bases, hydrogen bond donors and acceptors were incorrectly placed on the purine bases to analyze the origins of discrimination between cognate and non-cognate RNA. The results of these experiments show that three different approaches are used by the U1A protein to gain specificity for purines. Specificity for the first base in the loop, A1, is based primarily on discrimination against RNA containing the incorrect base, specificity for the fourth base in the loop, G4, is based largely on recognition of the donors and acceptors of G4, while specificity for the sixth base in the loop, A6, results from a combination of direct recognition of the base and discrimination against incorrectly placed functional groups.

**Conclusion:**

These investigations identify different roles that hydrogen bond donors and acceptors on bases in both cognate and non-cognate RNA play in the specific recognition of RNA by the U1A protein. Taken together with investigations of other RNA-RRM complexes, the results contribute to a general understanding of the origins of RNA-RRM specificity and highlight, in particular, the contribution of steric and electrostatic repulsion to binding specificity.

## Background

The RRM is one of the most common RNA-binding domains [1-3] and is found in proteins that participate in all steps of gene expression and RNA processing [4,5]. The RRM is approximately 100 amino acids and forms a general single-stranded RNA binding scaffold comprised of a four-stranded anti-parallel β-sheet flanked by two α-helices [6]. RRMs bind RNAs of different sequences and in many different structural contexts. In general, RRMs make limited contacts with the sugar-phosphate backbone compared to other RNA-binding proteins and large cooperative networks of hydrogen bonds are formed with the nucleobases. Although individual structures of RRM-RNA complexes have been solved [7,8], it remains unclear how this domain forms a general RNA binding scaffold, while individual proteins containing RRMs achieve high specificity for particular RNA sequences.

**Figure 1:**
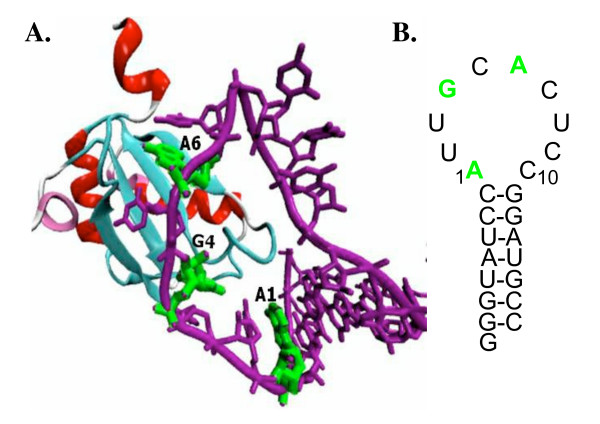
**A**. Diagram of the U1A-SL2 RNA complex from the X-ray cocrystal structure [16]. A1, G4, and A6 are displayed in green. **B. **Stem loop 2 of U1 snRNA.

Extensive biophysical and biochemical investigations have made U1A a paradigm of RRM-RNA recognition [9-22]. U1A is a component of the U1 snRNP and also regulates polyadenylation of U1A pre-mRNA [23-25]. The N-terminal RRM of U1A binds with high affinity to stem loop 2 (SL2) in U1 snRNA and an internal loop target site of nearly identical sequence in the U1A pre-mRNA [9,26]. The structure of the N-terminal RRM of U1A bound to SL2 RNA is shown in Figure 1[16]. U1A contacts the AUUGCAC sequence of the loop and the closing CG base pair. Most hydrogen bond donors and acceptors of these bases are involved in hydrogen bonds with protein main chain or side chain functional groups in loop 1, loop 3, β1, β4, and loop 6 of U1A. In addition, conserved aromatic amino acid side chains in β1 and β3 stack with RNA bases C5 and A6. Cooperative networks of interactions important for complex formation involving amino acids in loops 1, 3, and 6 and β1, β3, and β4 have been identified experimentally and have been suggested computationally [13,15,18,22,27-31].

We are using the U1A-RNA complex as a model system to understand how RRMs are modulated for specific recognition of RNA target sites [15,18,19,21,22]. The U1A protein is highly specific for the AUUGCAC loop sequence. The AUUGCAC sequence was selected for binding to U1A with 87–100% conservation at each site in a variety of loop sizes or in a linear sequence [26]. The U1A protein is particularly sensitive to base substitutions for the purines in the RNA target site. Mutation of A1 to G resulted in a large destabilization of the complex of 5.0 kcal/mol (Table 1) [9,28]. Mutation of the purines G4 or A6 to A or G, respectively, resulted in a 50,000–100,000-fold increase in the *K*_D _of the complex and a 6.4–6.8 kcal/mol destabilization of the complex (Tables 2 and 3) [18,28]. Mutations of pyrimidines in the target sequence were less destabilizing, 1.6–4 kcal/mol [9].

**Table 1:** Stability of complexes of wild type U1A protein with SL2 RNA sequences containing A1 modifications.

	WT	A1P	A1c1A	A12AP	A1DAP	A1I	A1G
*K*_D _(M)^a^	3 ± 2 × 10^-10^	9 ± 2 × 10^-10^	7 ± 2 × 10^-10^	4 ± 1 × 10^-8^	4 ± 2 × 10^-8^	1.6 ± 0.1 × 10^-8^	1.4 ± 0.4 × 10^-6^
ΔG (kcal/mol)^b^	-13.0 ± 0.4	-12.3 ± 0.4	-12.5 ± 0.2	-10.1 ± 0.1	-10.1 ± 0.3	-10.6 ± 0.02	-8.0 ± 0.1
ΔΔG^c^		0.7	0.5	2.9	2.9	2.4	5.0

^a^*K*_D _values are the average of at least three independent experiments. Standard deviations determined from the independent measurements are reported. ^b^ΔG is the free energy of association of the complex. ^c^ΔΔG is the difference between the binding energies of the wild type U1A protein for SL2 RNA containing the indicated base substitution and wild type SL2 RNA.

**Table 2:** Stability of complexes of wild type U1A protein with SL2 RNA sequences containing G4 modifications.

	WT	G4dG	G4c7dG	G4I	G42AP	G4DAP	G4A
*K*_D _(M)^a^	3 ± 2 × 10^-10^	1.1 ± 0.4 × 10^-10^	3.1 ± 0.1 × 10^-8^	3 ± 1 × 10^-9^	4 ± 1 × 10^-6^	2 ± 1 × 10^-6^	1.1 ± 0.2 × 10^-5^
ΔG (kcal/mol)^b^	-13.2 ± 0.4	-13.6 ± 0.2	-10.3 ± 0.01	-11.7 ± 0.2	-7.4 ± 0.1^e^	-7.8 ± 0.3	-6.8 ± 0.1^e^
ΔΔG^c^		-0.4	3.3^d^	1.5	5.8	5.4	6.4

^a^*K*_D _values are the average of at least three independent experiments. Standard deviations determined from the independent measurements are reported. ^b^ΔG is the free energy of association of the complex. ^c^ΔΔG is the difference between the binding free energies of the wild type U1A protein for SL2 RNA containing the indicated base substitution and wild type SL2 RNA. ^d^ΔΔG is the difference between the binding free energies of the wild type U1A protein for SL2 RNA containing the indicated base substitution and SL2 RNA containing G4dG. ^e ^These binding curves did not reach saturation. Thus, the binding constants given were estimated by assuming saturation would be reached at 70–100% bound as discussed in the experimental section.

**Table 3:** Stability of complexes of wild type U1A protein with SL2 RNA sequences containing A6 modifications.

	WT	A6P	A6c1A	A62AP	A6DAP	A6I	A6G
*K*_D _(M)^a^	3 ± 2 × 10^-10^	1 × 10^-8c^	1.2 × 10^-8d^	7 ± 1 × 10^-7^	1.1 ± 0.4 × 10^-7^	8 ± 2 × 10^-6^	3 × 10^-5d^
ΔG (kcal/mol)^b^	-13.0 ± 0.4	-10.9^d^	-10.8^d^	-8.4 ± 0.1	-9.5 ± 0.3	-6.9 ± 0.2	-6.2^d^
ΔΔG^c^		2.1	2.2	4.6	3.5	6.1	6.8

^a^*K*_D _values are the average of at least three independent experiments. Standard deviations determined from the independent measurements are reported. ^b^ΔG is the free energy of association of the complex. ^c^ΔΔG is the difference between the binding energies of the wild type U1A protein for SL2 RNA containing the indicated base substitution and wild type SL2 RNA. ^d ^Previously reported in ref. 18.

We have focused on investigating the specificity of U1A for the purines A1, G4, and A6 in the target SL2 RNA, because U1A is finely tuned to recognize the correct purine bases at these positions. The data we report here suggest that for A1, discrimination against non-cognate RNA is a significant contributor to specificity in the absence of substantial direct contacts between U1A and the base. In contrast, hydrogen bond donors and acceptors on G4 are essential contributors to binding and cannot be substituted with aliphatic groups. Recognition of A6 involves both positive contributions to binding by A6 hydrogen bond donors and acceptors and the destabilization of complexes with incorrectly placed functional groups. Thus, the relative contributions to specificity of hydrogen bond donors and acceptors on cognate and non-cognate RNA bases vary for recognition of A1, G4, and A6 in SL2 RNA by the U1A protein.

## Results

### Strategy for Probing Specificity

To investigate the specificity requirements of the U1A protein, we have measured the affinity of the U1A protein for SL2 RNA target sites containing modified purine bases. In one series of modifications, individual hydrogen bond donors or acceptors were eliminated or substituted with aliphatic groups to probe the energetic contributions of these functional groups to binding. In a second series of modifications, the purines were substituted with alternative hydrogen bond donors or acceptors to investigate the ability of the U1A protein to discriminate against incorrectly placed functional groups. It should be noted that these experiments do not identify the molecular origins of changes in binding affinity observed when altering a hydrogen bond donor or acceptor in the complex. The effects of these base modifications on binding are likely to be complex because they may alter the complex interface, change the structure and dynamics of both the free RNA and the complex, alter cooperative networks of interactions involved in binding, or change solvation effects. The experiments reported here probe the importance of selected functional groups to the specificity of binding, and this importance may arise by altering any or all of these contributions to binding affinity.

### Recognition of A1

**Figure 2:**
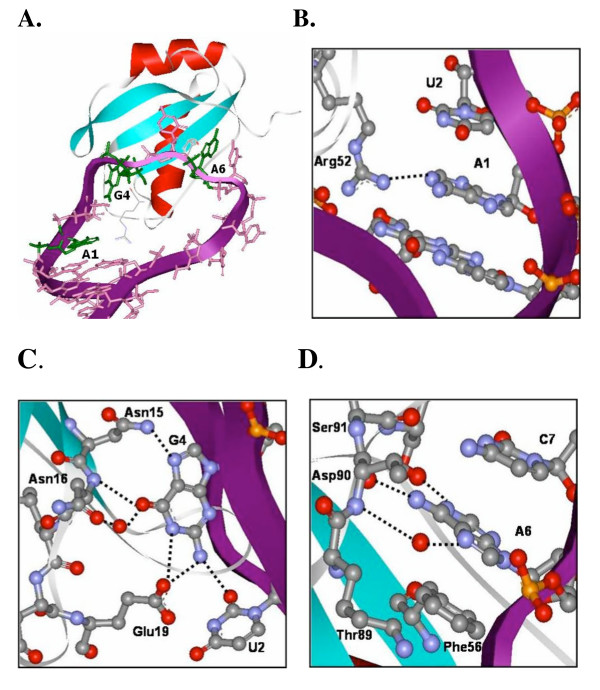
The interaction of U1A and SL2 RNA. **A**. Diagram of the U1A-SL2 RNA complex from the X-ray cocrystal structure [16]. A1, G4, and A6 are displayed in green. Panels **B**, **C**, and **D **show an expanded view of the hydrogen bonding network involving A1, G4, and A6, respectively, in the cocrystal structure.

**Figure 3:**
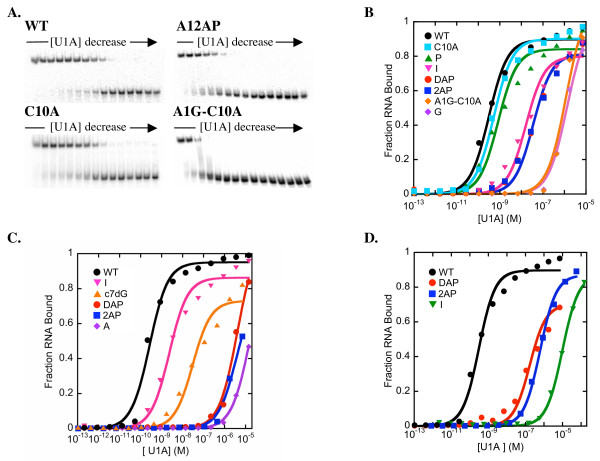
**A. **Representative gel mobility shift analysis of U1A protein binding to WT, A1-2AP, C10A, and A1G-C10A SL2 RNAs. The highest protein concentration used for the assays was 7 μM, and a 1:3 serial dilution was performed. **B.-D. **Plots illustrating the fraction RNA bound as a function of U1A concentration. **B. **Binding experiments performed with SL2 RNAs in which A1 was substituted with P, I, DAP, 2AP, and G. Binding curves for C10A and A1G-C10A are also included in this plot. **C. **Binding experiments performed with SL2 RNAs in which G4 was substituted with I, c7dG, DAP, 2AP, and A. **D. **Binding experiments performed with SL2 RNAs in which A6 was substituted with DAP, 2AP, and I.

A1 stacks between U2 and the CG base pair that closes the loop in the U1A-SL2 RNA complex (Figure 2A, 2B) [16]. A1 forms only one hydrogen bond with the U1A protein, which is between N1 and the side chain of Arg52. The side chain of Arg52 also forms hydrogen bonds with the G that forms the closing GC base pair of SL2 RNA. Substitution of Arg52 with Gln abolishes binding [11,32]. The *K*_D_'s of complexes formed with SL2 RNAs containing modifications at the A1 position are reported in Table 1. Representative gel mobility shift analyses and binding curves are shown in Figure 3. Despite the small number of hydrogen bonds between A1 and the U1A protein, the substitution of G for A1 resulted in a 5 kcal/mol destabilization of the complex. This is similar to the destabilization of the complex observed previously for the substitution of A1 with C [9,28]. The substitution of G for A1 may introduce a base pair between G and C10 on the 3'-end of the loop, thus reducing the loop to 8 nucleotides. This additional base pair would be expected to destabilize the complex because complex formation would require dissociation of the additional base pair. However, the double mutant A1G C10A SL2 RNA, which cannot form an analogous additional base pair, bound with similar affinity (*K*_D _= 1.3 ( ± 0.6) × 10^-6 ^M) as A1G SL2 RNA to U1A. The C10A substitution alone had little effect on binding affinity (*K*_D _= 6 ( ± 1) × 10^-10 ^M). Thus, these experiments suggest that specific recognition of A1 does not require an extensive hydrogen bond network with the base.

To probe the role of base functional groups without introducing new functional groups A1 was substituted with purine, in which the 6-NH_2 _group is replaced with hydrogen, and with c1A in which N1 is replaced with C-H. Both substitutions resulted in very small destabilizations of the complex (0.5–0.7 kcal/mol) even though a hydrogen bond is formed between N1 and the side chain of Arg52 in the X-ray structure. In contrast, base substitutions that altered the pattern of hydrogen bond donors and acceptors, such as A1I, or added hydrogen bond donors or acceptors, such as A1DAP or A1-2AP, resulted in a much larger destabilization of the complex of 2.4–2.9 kcal/mol. Within the wild type structure the 2AP and DAP substitutions would introduce unfavorable steric interactions between the side chain of Leu49 and the 2-NH_2 _group, while the substitution of inosine for A1 would introduce an electrostatic repulsion between N1-H and the side chain of Arg52. These unfavorable interactions may contribute to the destabilization of the complexes containing A1-2AP and A1-DAP, which could contribute to alternate structures being formed in these complexes. These data suggest that the poor affinity of A1G SL2 RNA for U1A is due to the altered pattern of hydrogen bond donors and acceptors on G compared to A, rather than a loss of specific hydrogen bonding interactions. Thus, at this position, specificity is a consequence primarily of the destabilization of complexes containing non-cognate bases.

### Recognition of G4

G4 is packed between U3 and a number of amino acids in the U1A protein (Figure 2C) [16]. G4 forms the largest number of hydrogen bonds with the U1A protein of any base in the AUUGCAC sequence. Hydrogen bonds are formed between N7 and the amide side chain of Asn15, O6 and the main chain amide of Asn16, O6 and the main chain carbonyl of Leu17 (water-mediated), 2-NH_2 _and O2 of U2, 2-NH_2 _and the side chain of Glu19, and N1-H and the side chain of Glu19. Substitution of A for G4 resulted in a 6.4 kcal/mol loss of binding affinity. This value is comparable to that reported previously [28].

The binding affinities of U1A for SL2 RNAs containing a series of base analogs substituted for G4 are reported in Table 2. Representative gel mobility shift analyses and binding curves are shown in Figure 3. The elimination of 2-NH_2 _(G4I) or the substitution of N7 with C-H (G4c7G) resulted in destabilizations of the complex of 1.5 and 3.3 kcal/mol, respectively. The greater loss in binding free energy upon substitution of G4 with c7G than with I suggests that N7 is a more important contributor to binding than is the 2-NH_2 _group even though two hydrogen bonds are observed between the 2-NH_2 _group and the U1A protein in the X-ray structure, compared to one hydrogen bond between N7 and the U1A protein. \These results are consistent with previously performed protein substitutions. The substitution of Asn15, which forms a hydrogen bond between the amide side chain and N7 of G4, with Val abolished binding [11]. In contrast, the substitution of Glu19, which forms a hydrogen bond between the side chain and 2-NH_2_, with Ser destabilized the complex, but did not abolish binding [33].

The substitution of either 2AP or DAP for G4 results in a larger destabilization of the complex, 5.8 and 5.4 kcal/mol, respectively, than was observed for either the G4I or G4c7G substitutions, perhaps because these substitutions alter functionality at both the 1 and 6 positions of the purine ring. Because of the weak binding affinity of U1A for these RNA sequences full binding curves were not obtained leading to more uncertainty in the reported dissociation constants compared to those for other RNA sequences. However, the large destabilization of the complex with the G4-2AP substitution suggests that the 6-O or N1-H groups are essential for binding to U1A. In the structure of the wild type complex G4DAP and G4-2AP substitutions would introduce an electrostatic repulsion between the lone pair of N1 and the side chain of Glu19. However, based on the low binding affinity of U1A for SL2 RNA containing these substitutions, it is likely that the structure of these complexes are altered from the wild type structure. The significant destabilization of the complex observed upon substitution of hydrogen bond donors and acceptors on G4 with aliphatic groups and the substitution of G4 with 2AP suggest that the specificity of the U1A protein for G4 is dependent in large part on direct and indirect contributions of the G4 functional groups to complex stability.

### Recognition of A6

The interactions between A6 and U1A in the X-ray cocrystal structure are shown in Figure 2D[16]. A6 stacks between Phe56 and C7. The substitution of non-aromatic amino acids for Phe56 results in a large destabilization of the complex [15,21,34]. N1 forms a hydrogen bond with the side chain of Ser91, the 6-NH_2 _forms a hydrogen bond with the main chain carbonyl of Thr89, and N7 forms a water-mediated hydrogen bond with the main chain amide of Thr89. We previously showed that the substitution of A with any other base results in a large destabilization of the complex of 6.3–6.7 kcal/mol, while the elimination of individual hydrogen bond donors or acceptors resulted in a 0.8–1.9 kcal/mol destabilization of the complex [18]. The substitution of Ser91 with Ala resulted in a similar destabilization of the complex as resulted from the substitution of N1 of A6 with a C-H group [19]. In addition, we observed energetic coupling between Phe56 and hydrogen bond donors and acceptors on A6 [18]. Thus, the hydrogen bond donors and acceptors on A6 play direct and indirect roles in stabilizing the complex.

The exchange of hydrogen bond donors and acceptors (for example, A6I) or the addition of a hydrogen bond donor (A6DAP) resulted in a much larger destabilization of the complex than the elimination of individual hydrogen bond donors and acceptors (Table 3, Figure 3). Within the context of the wild type structure, the introduction of the 2-NH_2 _would introduce unfavorable steric interactions with the side chain of Leu44, an amino acid previously suggested to be important for specific recognition of SL2 RNA [35], and the substitution of inosine for A6 would result in unfavorable steric interactions between N1-H and the side chain of Ser91 and an unfavorable electrostatic interaction between O6 and the main chain carbonyl of Asp90. These unfavorable interactions may destabilize the complex, which could contribute to alternative complex structures being formed upon incorporation of these modified bases. Together, the results from the experiments eliminating hydrogen bond donors and acceptors from A6 reported previously [18] and those exchanging hydrogen bond donor and acceptors reported here suggest that specific recognition of A6 involves both direct recognition of the base and discrimination against incorrectly placed functional groups.

### RNA Stability

The large destabilizations of the complexes formed with U1A upon incorporation of many of the base analogs described here suggests considerable variation in free RNA or complex structure as a result of these base substitutions. The RNA loop is dynamic when free, making it difficult to characterize the effect of the base substitutions for A1, G4, and A6 on RNA structure. Because the base analogs contain modified hydrogen bond donor and acceptor patterns that are similar in polarity and stacking ability to G and A, it is likely that the primary effect of the base analogs is to alter the structure of the complex. However, we were concerned that the A1G SL2 RNA and perhaps the A1I SL2 RNA could form an additional base pair, thus stabilizing SL2 RNA and reducing the size of the loop.

To investigate the effect of base substitutions on SL2 RNA stability, temperature dependent melting analyses were performed. The *T*_m_'s of the SL2 RNAs not previously reported are listed in Table 4. A limitation of this method is that it primarily probes the structure and stability of the helical portion of SL2 RNA, rather than the loop. Thus, not surprisingly, the *T*_m_'s of SL2 RNAs containing base substitutions were similar to or within error of that of the wild type RNA. The measured *T*_m_'s varied between 57°C and 62°C, with no correlation observed between the *T*_m _and the binding affinities reported in Tables 1, 2, 3. As expected, the *T*_m _of the A1G SL2 RNA (64.2 ± 0.5°C) was 2°C higher than the range observed for the other RNA's, including A1I SL2 RNA (61 ± 2°C). The *T*_m _of the SL2 RNA containing A1G/C10A, which eliminates the possibility of an additional GC base pair, was measured to be within the range of the other SL2 RNAs (61.6 ± 0.9). Because the stabilities of the complexes containing A1G and A1G/C10A SL2 RNAs are within experimental error and the C10A substitution does not itself alter the stability of the complex, the additional stability of the A1G SL2 RNA does not appear to be a large contributor to the destabilization of the complex by this substitution.

**Table 4:** Results of temperature dependent melting analyses of SL2 RNAs.

RNA	*T*_m_
Wild type	60.5 ± 0.6
A1P	59.5 ± 0.9
A1c1A	62 ± 1
A12AP	61 ± 2
A1DAP	62 ± 1
A1I	61 ± 2
A1G	64.2 ± 0.5
A1G/C10A	61.6 ± 0.9
G4c7dG	56.8 ± 0.6
G4I	60.1 ± 0.2
G42AP	59 ± 1
G4DAP	60 ± 2
G4A	58.9 ± 0.3
A62AP	60 ± 1
A6DAP	59.7 ± 0.5
A6I	60.6 ± 0.7

## Discussion

The substitution of the three essential purines in SL2 RNA with bases in which hydrogen bond donors and acceptors are replaced with aliphatic groups and with bases in which hydrogen bond donors and acceptors are placed incorrectly on the base has enabled us to compare the positive contributions of correct functional groups with the negative contributions of incorrectly placed functional groups to the binding specificity of the U1A protein. These comparisons have suggested that specific recognition of essential purines by the U1A protein varies from primarily discrimination against non-cognate bases for A1 to direct contributions of the base functional groups for G4. The base modifications introduced in these experiments are likely to not only eliminate and introduce individual interactions that either destabilize or stabilize the complex, but to affect other interactions in the complex that are energetically coupled with the modified base. Cooperative networks of interactions involving both protein and RNA residues have been identified experimentally and suggested computationally in the U1A system [13,18,22,27-31]. These studies have focused more on cooperative networks in the free and bound proteins than on networks involving RNA. However, experiments have suggested energetic coupling between A6 and Phe56 and between loop 3 of U1A, Tyr13, Gln54 and the RNA [13,18], and an analysis of collective atomic fluctuations in MD simulations of the U1A-SL2 RNA complex suggest large networks of interactions involving both protein and RNA that have not yet been explored experimentally [22,30]. Thus, the energetic roles of individual hydrogen bond donors and acceptors that we have identified to be important for the discrimination by U1A between cognate and non-cognate RNA sequences are likely to be complex.

Previously, Shamoo and coworkers investigated the recognition of purines by two linked RRMs from hnRNPA, called UP1 [36]. UP1 is a significantly different RRM than U1A and a comparison of U1A and UP1 should be valuable for developing principles of RRM-RNA specificity. Shamoo and coworkers suggested three generalizations of RNA recognition by RRMs based on experiments with UP1. First, interactions with main chain amides might provide greater base discrimination than interactions with side chains. Although this idea has not yet been fully evaluated in the U1A-RNA complex, binding studies that have been performed with RNAs containing modified bases have suggested that interactions with main chain amides are not necessarily more important than those with side chains in the U1A-RNA complex. For example, N7 of G4 forms a hydrogen bond with the side chain of Asn15. As reported in Table 2, substitution of N7 with C-H results in a significant destabilization of the complex. In contrast, the substitution of A6 with purine, which eliminates the 6-NH_2 _group that forms a hydrogen bond with the main chain carbonyl of Thr89 destabilizes the complex comparably as the substitution of N1, which forms a hydrogen bond with the side chain of Ser91, with C-H [15,19]. Second, hydrogen bonds to bases involving charged amino acids are more energetically important than those involving neutral amino acid functional groups. U1A does not have a large number of hydrogen bonds with charged residues, so these are not required for tight binding. Third, steric repulsion is a key discriminatory tool for gaining sequence specificity. In fact, for the U1A-RNA complex, steric and electrostatic repulsion due to incorrectly placed functional groups can be more important than direct base recognition in controlling specificity. Thus, specific recognition of RNA target sites by UP1 and U1A are similarly guided by discrimination against non-cognate RNA.

## Conclusion

In conclusion, these investigations show three different approaches used by the U1A protein to specifically recognize essential purines in the SL2 RNA target site and underscore the ability of steric and electrostatic repulsion to be important for specificity even in the absence of a direct hydrogen bond network with the base. The contributions of negative recognition to specificity have been shown to be important in RNA recognition by other proteins, for example by the MS2 coat protein and tRNA synthetases, and also in DNA-protein recognition [37-45]. Thus, the data from the U1A-SL2 RNA and UP1-RNA complexes extend this generalization to the RRM-RNA complexes. Together, these results form part of a growing body of data that shows the importance of steric and electrostatic discrimination against incorrectly placed functional groups on non-cognate bases in governing the specificity of RNA-protein complexes. These general principles describing the origins of specificity of protein-RNA complexes will be invaluable in understanding and controlling complex formation.

## Methods

### Protein Expression and Purification

An expression vector for the N-terminal RRM of U1A (amino acids 1–102) was obtained from Nagai [11]. The wild type protein was expressed and purified as described previously [18]. The molecular weight was confirmed by ESI mass spectrometry and the concentration was determined by amino acid analysis.

### RNA Synthesis and Purification

RNA containing c7G was synthesized at the KECK facility at Yale Medical School. Other oligonucleotides were purchased from Dharmacon and IDT. The RNA and chimeric oligomers were purified using denaturing gel electrophoresis. Concentrations were determined by UV at 260 nm. All oligonucleotides were characterized by MALDI mass spectrometry.

### Equilibrium Binding Assays

Protein-RNA equilibrium dissociation constants were measured by gel mobility shift assays. Reaction mixtures containing 25 pM ^32^P-labeled RNA and protein in 10 mM Tris-HCl (pH 7.4), 250 mM NaCl, 1 mM EDTA, 0.5% Triton X-100 and 1 mg/mL tRNA in a total volume of 14 μL were equilibrated for at least 45 min. After addition of glycerol to a final concentration of 5%, the reactions were separated on an 8% polyacrylamide gel in a buffer containing 100 mM Tris-borate (pH 8.3), 1 mM EDTA, and 0.1% Triton X-100 for 35 min at 350 V. The temperature of the gel was maintained at 25°C by a circulating water bath. Gels were analyzed on a Molecular Dynamics Storm Phosphorimager. Fraction RNA bound versus protein concentration was plotted and curves were fit to the equation: fraction bound = 1/(1+*K*_D_/[P]), where [P] is the total protein concentration. In gel mobility shift assays that did not saturate at approximately 100% bound, this equation was modified to: fraction bound = A/(1+*K*_D_/[P]), where A was allowed to vary between 0.7 and 1. This modification was necessary to estimate the *K*_D_'s of some of the least stable complexes. Representative gel mobility shift assays and plots illustrating fraction RNA bound as a function of U1A concentration are shown in Figure 3. The *K*_D_'s obtained from these experiments are listed in Tables 1, 2, 3. The errors listed in the tables are the standard deviations of the results of at least three independent binding experiments and thus, represent the reproducibility of the experimental data.

### RNA Melting Experiments

UV melting curves were performed on a Shimadzu UV-2401PC spectrophotometer using 2–10 μM RNA samples in 100 mM NaCl, 0.5 mM EDTA, and 10 mM sodium phosphate at pH 7. The samples were heated from 10 to 95 °C with a heating rate of 1 °C/min, while monitoring absorbance at 280 nm. The melting curves were fit using the program Meltwin 3.5.

## Authors' contributions

YB carried out the all of the experiments presented here and participated in the design of the study. AMB conceived of the study, participated in its design and coordination, and drafted the manuscript. Both authors have read and approved the final manuscript.
